# Younger patients with colorectal cancer may have better long-term survival after surgery: a retrospective study based on propensity score matching analysis

**DOI:** 10.1186/s12957-024-03334-4

**Published:** 2024-02-21

**Authors:** Weihao Liao, Yuanxi Li, Yuheng Zou, Qinchen Xu, Xiaodong Wang, Li Li

**Affiliations:** 1 Department of General Surgery, Division of Gastrointestinal Surgery, West China Hospital, Sichuan University, Chengdu, Sichuan China; 2 West China School of Medicine, Sichuan University, Chengdu, Sichuan China; 3 Gastric Cancer Center, West China Hospital, Sichuan University, Chengdu, Sichuan China; 4 Colorectal Cancer Center, West China Hospital, Sichuan University, Chengdu, Sichuan China

**Keywords:** Colorectal cancer, Younger, Older, Propensity score matching, Long-term survival

## Abstract

**Background:**

The relationship between postoperative long-term prognosis and age in colorectal cancer patients remains controversial. The purpose of this study based on a Chinese CRC cohort is to determine the disparity in long-term survival outcomes between younger and older colorectal cancer (CRC) patients after surgery using a propensity score matching (PSM).

**Methods:**

Data for this study was derived from the CRC cohort of the Database from Colorectal Cancer (DACCA) at West China Hospital of Sichuan University from January 2007 to September 2022. The long‑term prognoses were compared between younger and older groups.

**Results:**

A total of 2374 CRC patients were evaluated in this study, including 1039 older patients and 1335 younger ones. After 1:1 ratio PSM, each group contained 784 CRC patients. There was no significant difference in baseline information after PSM (*p* < 0.05). Multivariate analysis showed that younger age was an independent predictor of better overall survival (OS) (*p* < 0.001, *HR* = 1.750, 95% *CI* = 1.407–2.177) and disease-specific survival (DSS) (*p* < 0.001, *HR* = 1.718, 95% *CI* = 1.369–2.157). In terms of different tumor pathological stages after PSM, in comparison to older group, younger group had better OS in stage II (*p* < 0.001), stage III (*p* = 0.0085), and stage IV (*p* = 0.0014) and better DSS in stage II (*p* = 0.0035), stage III (*p* = 0.0081), and stage IV (*p* < 0.001).

**Conclusion:**

Younger CRC patients have better prognosis than older CRC patients after surgery, especially, and have better OS and DSS in stages II, III, and IV CRC. Younger CRC patient may gain greater benefit from CRC resection and combined therapy. As for the cut-off age, it may be determined by a specific model suitable for local patients.

## Introduction

Colorectal cancer (CRC) ranks third in global morbidity and second in mortality [1]. The International Agency for Research on Cancer (IARC) estimated that in 2020, over 1.9 million new CRC cases emerged, with more than 930,000 CRC-related fatalities [2]. China is home to approximately one-fifth of the global population. With the rapid societal development, cancer, like other chronic diseases, has become a prominent burden on China’s populace. Statistical analysis reveals that between 1990 and 2015, age-standardized incidence and mortality rates of CRC in China exhibited an upward trend [3]. In 2020, China’s new CRC cases and fatalities constituted 29.54% and 29.26% of the worldwide totals, respectively [4, 5]. Furthermore, the incidence of young-onset CRC (age < 50 years) has risen in East Asia, including China, South Korea, and Japan from 1995 to 2014, suggesting that this trend is not exclusive to Western nations [6]. The same problem has arisen in India and in South Asia since 1994 [7]. Treatment and prognosis disparities among CRC patients of different ages have garnered considerable attention in recent years.

Presently, the prognosis of CRC patients across different age groups remains a contentious topic. Some studies have reported worse outcomes for younger CRC patients compared to their older counterparts [8, 9], whereas others have indicated better outcomes for the younger demographic [10] or comparable survival rates between the two age groups [11]. These discrepancies may arise from differing definitions of younger patients across studies. Some researchers designate patients ≤ 50 years old as younger [12], while others classify those ≤ 45 years old as such [13]. Inconsistent age groupings may lead to incongruous analytical outcomes, rendering a more objective statistical approach necessary—one that calculates and adjusts the cut-off age according to the data characteristics of the CRC cohort.

Moreover, due to inherent age differences, younger and older CRC patients will inevitably exhibit variations in specific variables, thereby introducing selection bias in the analysis of long-term prognoses among diverse age groups. Consequently, propensity score matching (PSM), a popular methodology in recent years, can be employed to balance the data between groups, yielding more accurate results in retrospective analyses.

In this study, we analyzed data from a Chinese CRC cohort by employing grouping and comparison methods following statistical processing. Our aim was to compare the long-term prognoses of younger and older CRC patients, examine the disparities in long-term survival among patients of different ages, and further investigate the most appropriate cut-off age. This study may be meaningful for exploring the differences in the long-term benefits of colorectal cancer resection for patients of different ages and guiding their surgical decisions.

## Method

### Data source

The data for this study is derived from the CRC cohort of the Database from Colorectal Cancer (DACCA) at West China Hospital of Sichuan University. The cohort is founded on 27 years of data collection. According to the latest guidelines at that time, CRC patients admitted to our hospital received diagnosis and treatment, such as regular surgery, chemotherapy, or/and radiotherapy. Data extraction was executed prior to data mining and analysis for this study, with information obtained from January 2007 to September 2022 iterations of the cohort.

### Inclusion and exclusion criteria

**Fig. 1:**
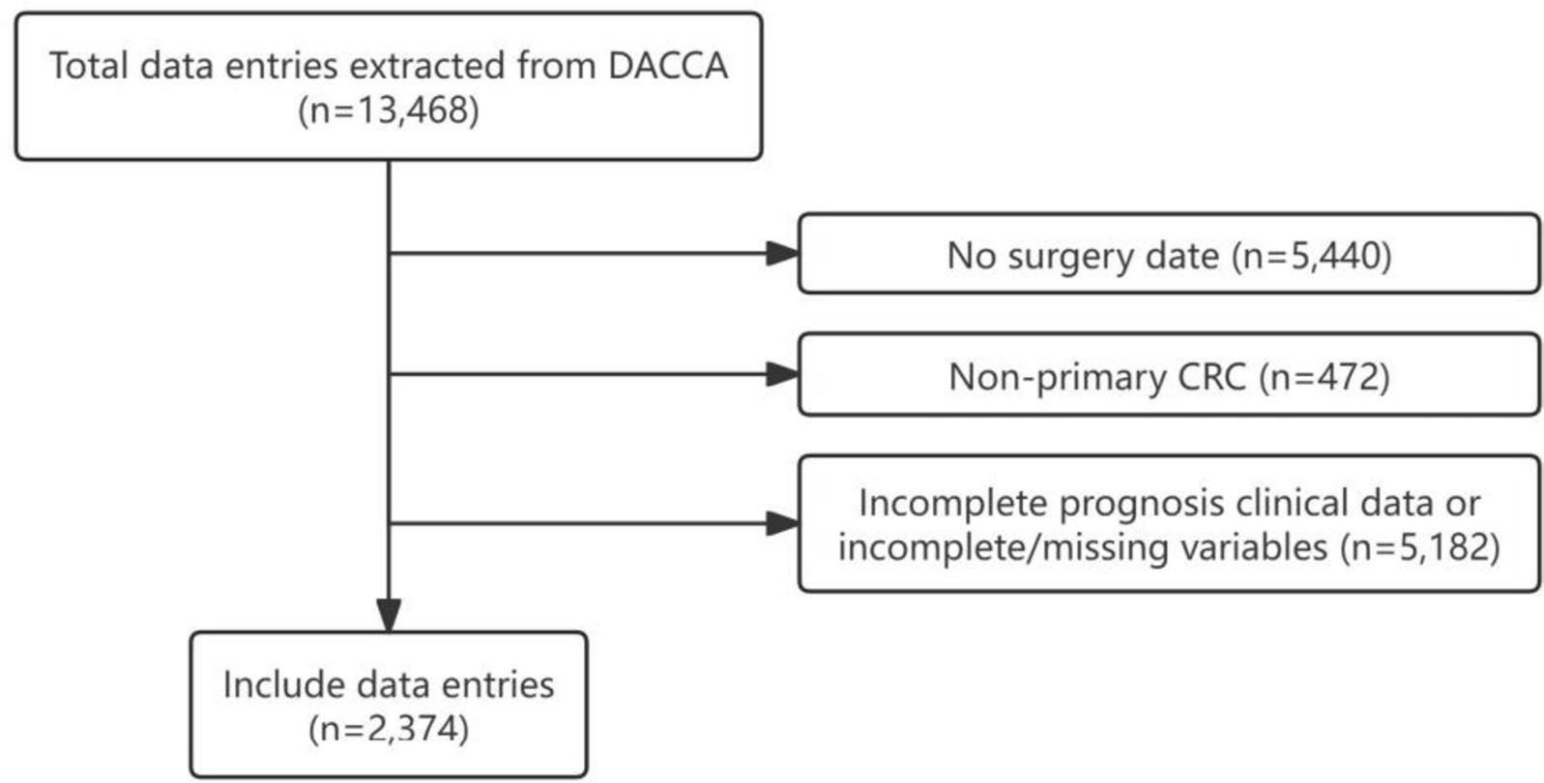
Flow chart of inclusion and exclusion

For this study, the inclusion criterion encompassed patients with a definitive diagnosis of CRC. The exclusion criteria incorporated patients who did not undergo CRC resection, patients with non-primary CRC, and patients with any incomplete prognosis clinical data or any one of incomplete/missing variables selected for this study. The inclusion and exclusion process is shown in Fig. 1.

The prognosis clinical data are necessary data to directly or indirectly generate the variables selected for analysis in this prognostic study, such as age and follow-up date. Taking into account the needs of research analysis and the existing data types that can be provided by DACCA, the variables used in this study were determined, involving demographics (age, sex, BMI), common age-related chronic diseases (diabetes mellitus, hypertension), and tumor pathology (tumor location, tumor differentiation, tumor pathological stage), treatment (surgical characteristic, adjuvant chemotherapy, adjuvant radiotherapy), and outcome (survival time, survival outcome). Survival time (months) was defined as the duration from the date of surgery to either the end of follow-up or the date of death and was employed for the analysis of overall survival (OS) and disease-specific survival (DSS). For the purposes of study analysis, tumor locations were classified into two categories: rectum and colon; surgical characteristics were classified into three categories: complete resection under the naked eye (R0), residual cancer under the microscope (R1), and residual cancer under the naked eye (R2); all kinds of tumor differentiation were classified into three categories: high, medium, and low differentiation.

### Age group division

Although converting continuous variables into categorical variables can lead to reduced statistical power and potential bias [14], categorization often facilitates interpretation and application, such as the categorization of body mass index (BMI) and ages in clinical practice. In this study, patients were required to be divided into younger and older groups based on their ages for analytical purposes. However, existing studies and guidelines employ varied age divisions. For instance, some guidelines advocate initiating screening for CRC from age 50 to 45 years [15, 16]. Given the inconvenience of directly referencing these divisions, we endeavored to identify the optimal cut-off point for dividing age groups using a novel approach.

The Cox proportional hazards regression model is a widely utilized survival prediction model in medicine for time-to-event data analysis [17]. Barrio et al. [18] described a method for classifying continuous predictor variables by employing this continuous predictor variable and time-to-event data in a Cox proportional hazards regression model to ascertain the optimal cut-off point. This method was implemented using the R function catpredi.survival. Consequently, we employed R (version 4.2.2) and the R function catpredi.survival in the R package CatPredi to determine the best cut-off age for OS and DSS outcome classifications, subsequently dividing age groups accordingly.

### PSM

Propensity score methods serve to mitigate or eradicate the influence of confounding variables when utilizing observational data, with PSM being one such method [19]. As a retrospective study, it is impossible to entirely eliminate the effects of confounding factor imbalances among varied control groups by randomizing subjects, as would be the case in a randomized controlled trial. Failure to address the imbalance of confounding factors will inevitably result in increased bias, ultimately compromising the validity of the study outcomes. In order to minimize these effects, we employed PSM to match the younger and older groups. Matching was executed based on confounding variables, encompassing sex, BMI, diabetes mellitus, hypertension, tumor location, tumor differentiation, tumor pathological stage, surgical characteristic, adjuvant chemotherapy, and adjuvant radiotherapy. The relevant procedures were implemented in R (version 4.2.2). Utilizing the function matchit in R and setting the clamp value at 0.03, we experimented with various matching ratios and determined the optimal ratio. Subsequently, the balance of the matched results was tested. A comparison of the baseline information between the younger and older groups was conducted before and after PSM.

### Statistical analysis

Before and after PSM, to ascertain differences in baseline characteristics between the younger and older cohorts, median tests were employed for BMI and age in both groups; chi-square tests were executed for additional variables, including sex, tumor location, tumor differentiation, tumor pathological stage, diabetes mellitus, hypertension, surgical characteristic, adjuvant chemotherapy, and adjuvant radiotherapy in both groups. A bilateral *P*-value of < 0.05 signified a statistically significant difference between the younger and older groups. After PSM, a univariate Cox regression analysis with age group as the independent variable, and a multivariate Cox regression analysis with age group and matched variables as independent variables, were conducted to identify independent predictors of better OS and DSS, which could evaluate the effect of age on the risk of postoperative mortality in CRC patients. After PSM, Kaplan–Meier curves were contrasted using log-rank tests for OS and DSS of different age groups, as well as for OS and DSS of different age groups in varied tumor pathological stages. *Z*-tests were employed for the comparison of OS and DSS across age groups at each time point. Bilateral *P*-values of < 0.05 were deemed statistically significant. The relevant procedures were implemented in R (version 4.2.2) or Statistical Product and Service Solutions (SPSS, version 26).

## Results

### Patients

In accordance with the established inclusion criteria, the CRC cohort provided a total of 13,468 data entries. Subsequently, the exclusion criteria were applied, resulting in the elimination of 5440 patients without recorded surgery dates, 472 patients with non-primary CRC, and 5182 patients with missing data in any of the inclusive parameters. Ultimately, a dataset consisting of 2374 patients was retained for subsequent analysis. See Fig. 1 for details.

### Age groups

Utilizing R obtained the optimal cut-off age which ascertained via the function catpredi.survival for OS and DSS outcome classifications, respectively. The derived optimal cut-off age for both classifications was 62.29 (retained two decimal places) years old. Consequently, the CRC patients were divided into a younger group (*n* = 1335) and an older group (*n* = 1039).

### PSM

PSM was employed to compare the younger group (*n* = 1335) and older group (*n* = 1039). Due to the presence of at least one statistically significant disparity following matching when the ratio exceeded 1, PSM was executed with a matching ratio of 1:1. The PSM results successfully passed the balance test, culminating in the formation of revised younger group (*n* = 784) and older group (*n* = 784).

Baseline characteristics of the younger and older CRC patient groups were compared before and after PSM. Before PSM, the younger group exhibited significant differences in diabetes mellitus (*p* < 0.001), hypertension (*p* < 0.001), tumor location (*p* = 0.028), tumor differentiation (*p* = 0.047), adjuvant chemotherapy (*p* < 0.001), and adjuvant radiotherapy (*p* = 0.009). After PSM, both groups comprised 784 patients, and no significant differences in baseline characteristic were observed (*p* > 0.05) (Table 1).

**Table 1:** Baseline characteristics before and after PSM

**Characteristics**	**Before PSM**			**After PSM**		
	Younger (1335)	Older (1039)	*p*-value	Younger (784)	Older (784)	*p*-value
**Age (year)**	52 (46.58)	69 (66.74)	< 0.001*	53 (46.59)	69 (65.74)	< 0.001*
**Sex**			0.191			0.757
**Male**	756 (56.6)	617 (59.4)		472 (60.2)	465 (59.3)	
**Female**	579 (43.4)	422 (40.6)		312 (39.8)	319 (40.7)	
**BMI (kg/m**^**2**^**)**	23.1 (20.4, 25.0)	22.7 (20.4, 24.6)	0.710	22.6 (20.3,25.0)	22.6 (20.2, 24.8)	0.960
**Diabetes mellitus**	177 (13.3)	263 (25.3)	< 0.001*	135 (17.2)	144 (18.4)	0.597
**Hypertension**	253 (19.0)	488 (47.0)	< 0.001*	243 (31.0)	240 (30.6)	0.913
**Tumor location**			0.028*			0.950
**Colon**	1082 (81.0)	803 (77.3)		625 (79.7)	623 (79.5)	
**Rectum**	253 (19.0)	236 (22.7)		159 (20.3)	161 (20.5)	
**Tumor differentiation**			0.047*			0.975
**Low**	392 (29.4)	259 (24.9)		205 (26.1)	207 (26.4)	
**Medium**	924 (69.2)	761 (73.2)		566 (72.2)	565 (72.1)	
**High**	19 (1.4)	19 (1.8)		13 (1.7)	12 (1.5)	
**Tumor pathological stage**			0.359			0.728
**0**	9 (0.7)	6 (0.6)		7 (0.9)	4 (0.5)	
**I**	156 (11.7)	120 (11.5)		84 (10.7)	87 (11.1)	
**II**	361 (27.0)	286 (27.5)		213 (27.2)	226 (28.8)	
**III**	408 (30.6)	282 (27.1)		228 (29.1)	210 (26.8)	
**IV**	401 (30.0)	345 (33.2)		252 (32.1)	257 (32.8)	
**Surgical characteristic**			0.075			0.430
**R2**	182 (13.6)	126 (12.1)		95 (12.1)	108 (13.8)	
**R1**	57 (4.3)	29 (2.8)		25 (3.2)	19 (2.4)	
**R0**	1096 (82.1)	884 (85.1)		664 (84.7)	657 (83.8)	
**Adjuvant chemotherapy**	419 (31.4)	224 (21.6)	< 0.001*	191 (24.4)	183 (23.3)	0.678
**Adjuvant radiotherapy**	37 (2.8)	12 (1.2)	0.009*	8 (1.0)	10 (1.3)	0.813

Continuous variables do not conform to a normal distribution and are expressed as median (lower quartile, upper quartile); categorical variables are expressed as *n* (%)

^*^P-value < 0.05

### Univariate and multivariate analysis of OS and DSS

In this study, regarding OS, older age (*p* < 0.001, *HR* = 1.750, 95% *CI* = 1.407–2.177), lower tumor differentiation (*p* < 0.001, *HR* = 1.463, 95% *CI* = 1.174–1.821), more advanced pathological stage (*p* < 0.001, *HR* = 1.827, 95% *CI* = 1.579–2.114), and poorer surgical characteristic (*p* < 0.001, *HR* = 1.609, 95% *CI* = 1.412–1.834) emerged as independent risk factors for OS (Table 2).

**Table 2:** Univariate and multivariate analysis of OS

**Risk factors**	**Univariate analysis**		**Multivariate analysis**	
	HR (95% CI)	*p*-value	HR (95% *CI*)	*p*-value
**Age (older/younger)**	1.596 (1.286, 1.982)	< 0.001*	1.750 (1.407, 2.177)	< 0.001*
**Sex (male/female)**	1.283 (1.028, 1.602)	0.028*	1.093 (0.872, 1.370)	0.440
**BMI**	0.977 (0.945, 1.010)	0.167	0.970 (0.940, 1.004)	0.080
**Diabetes mellitus (yes/no)**	1.270 (0.969, 1.665)	0.084	0.985 (0.732, 1.326)	0.922
**Hypertension (yes/no)**	1.148 (0.902, 1.460)	0.261	1.187 (0.909, 1.551)	0.208
**Tumor location (rectum/colon)**	1.044 (0.796, 1.371)	0.754	1.144 (0.868, 1.508)	0.341
**Tumor differentiation (low/medium/high)**	2.234 (1.814, 2.751)	< 0.001*	1.463 (1.174, 1.821)	< 0.001*
**Tumor pathological stage (0/I/II/III/IV)**	2.203 (1.924, 2.523)	< 0.001*	1.827 (1.579, 2.114)	< 0.001*
**Surgical characteristic (R2/R1/R0)**	2.140 (1.908, 2.400)	< 0.001*	1.609 (1.412, 1.834)	< 0.001*
**Adjuvant chemotherapy (yes/no)**	1.190 (0.910, 1.557)	0.204	0.889 (0.665, 1.190)	0.429
**Adjuvant radiotherapy (yes/no)**	2.301 (1.087, 4.874)	0.030*	0.912 (0.416, 1.997)	0.818

^*^P-value < 0.05

Pertaining to DSS, older age (*p* < 0.001, *HR* = 1.718, 95% *CI* = 1.369–2.157), lower tumor differentiation (*p* < 0.001, *HR* = 1.522, 95% *CI* = 1.211–1.913), more advanced tumor pathological stage (*p* < 0.001, *HR* = 2.071, 95% *CI* = 1.762–2.434), and poorer surgical characteristic (*p* < 0.001, *HR* = 1.578, 95% *CI* = 1.382–1.801) were identified as independent risk factors for DSS (Table 3).

**Table 3:** Univariate and multivariate analysis of DSS

Risk factors	Univariate analysis		Multivariate analysis	
	HR (95% *CI*)	*p*-value	HR (95% *CI*)	*p*-value
**Age (older/younger)**	1.540 (1.229, 1.929)	< 0.001*	1.718 (1.369, 2.157)	< 0.001*
**Sex (male/female)**	1.249 (0.992, 1.573)	0.059	1.041 (0.823, 1.318)	0.736
**BMI**	0.987 (0.954, 1.021)	0.451	0.982 (0.947, 1.017)	0.307
**Diabetes mellitus (yes/no)**	1.228 (0.923, 1.635)	0.159	0.934 (0.684, 1.276)	0.668
**Hypertension (yes/no)**	1.106 (0.858, 1.425)	0.437	1.078 (0.808, 1.438)	0.384
**Tumor location (rectum/colon)**	0.996 (0.753, 1.318)	0.978	1.103 (0.829, 1.467)	0.502
**Tumor differentiation (low/medium/high)**	2.392 (1.924, 2.973)	< 0.001*	1.522 (1.211, 1.913)	< 0.001*
**Tumor pathological stage (0/I/II/III/IV)**	2.538 (2.182, 2.952)	< 0.001*	2.071 (1.762, 2.434)	< 0.001*
**Surgical characteristic (R2/R1/R0)**	2.236 (1.986, 2.517)	< 0.001*	1.578 (1.382, 1.801)	< 0.001*
**Adjuvant chemotherapy (yes/no)**	1.298 (0.986, 1.708)	0.063	0.974 (0.723, 1.312)	0.862
**Adjuvant radiotherapy (yes/no)**	2.521 (1.189, 5.344)	0.016*	0.923 (0.420, 2.025)	0.841

^*^P-value < 0.05

### Differences in survival by age group

**Fig. 2:**
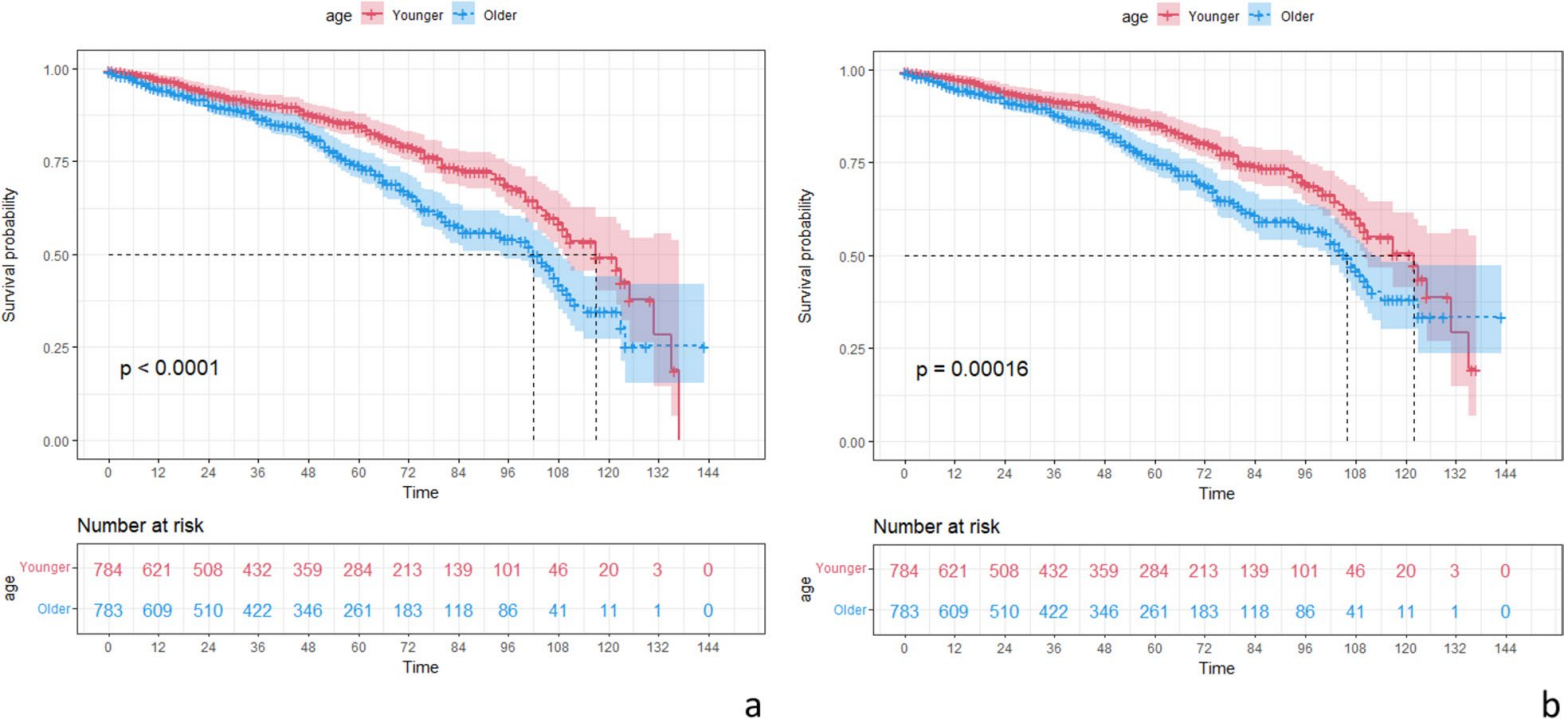
OS and DSS curves of different age groups. **a** OS curve. **b** DSS curve

**Fig. 3:**
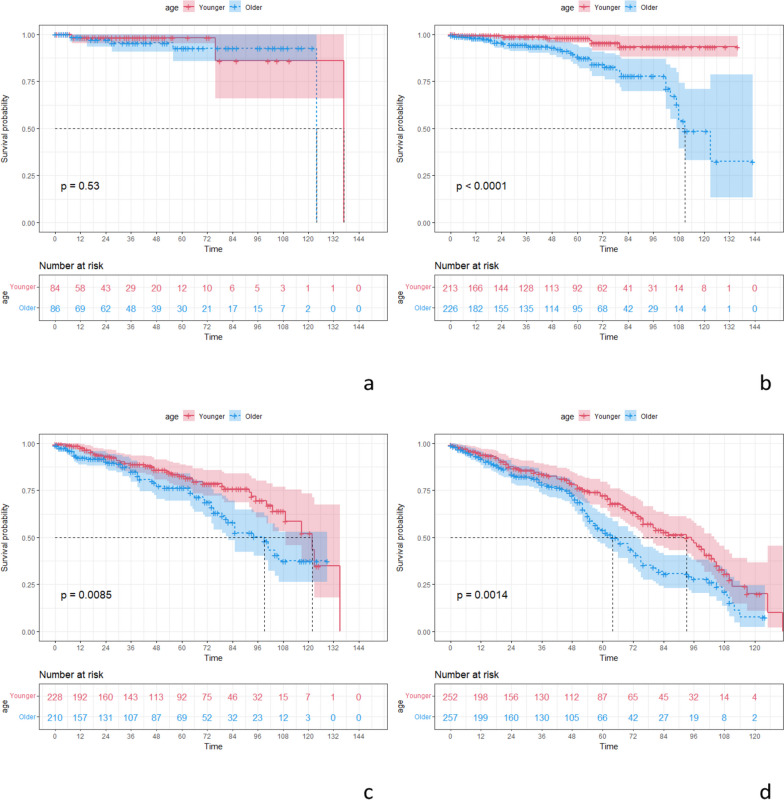
OS curves of different age groups in different tumor pathological stages. **a** Stage I. **b** Stage II. **c** Stage III. **d** Stage IV

After PSM, the younger group had better OS compared to the older group (Fig. 2). Regarding OS at varied time points, the younger group had better 1-year OS (*p* = 0.034), 3-year OS (*p* = 0.039), 5-year OS (*p* < 0.001), and 10-year OS (*p* = 0.035) relative to the older group. In terms of OS in different tumor pathological stages, the younger group had better OS in stage II (*p* < 0.001), stage III (*p* = 0.0085), and stage IV (*p* = 0.0014) compared to the older group (Fig. 3). For stage II CRC, the younger group had better 3-year OS (*p* = 0.031), 5-year OS (*p* = 0.002), and 10-year OS (*p* < 0.001); for stage III CRC, the younger group had better 1-year OS (*p* = 0.029); and for stage IV CRC, the younger group had better 5-year OS (*p* = 0.001).

**Fig. 4:**
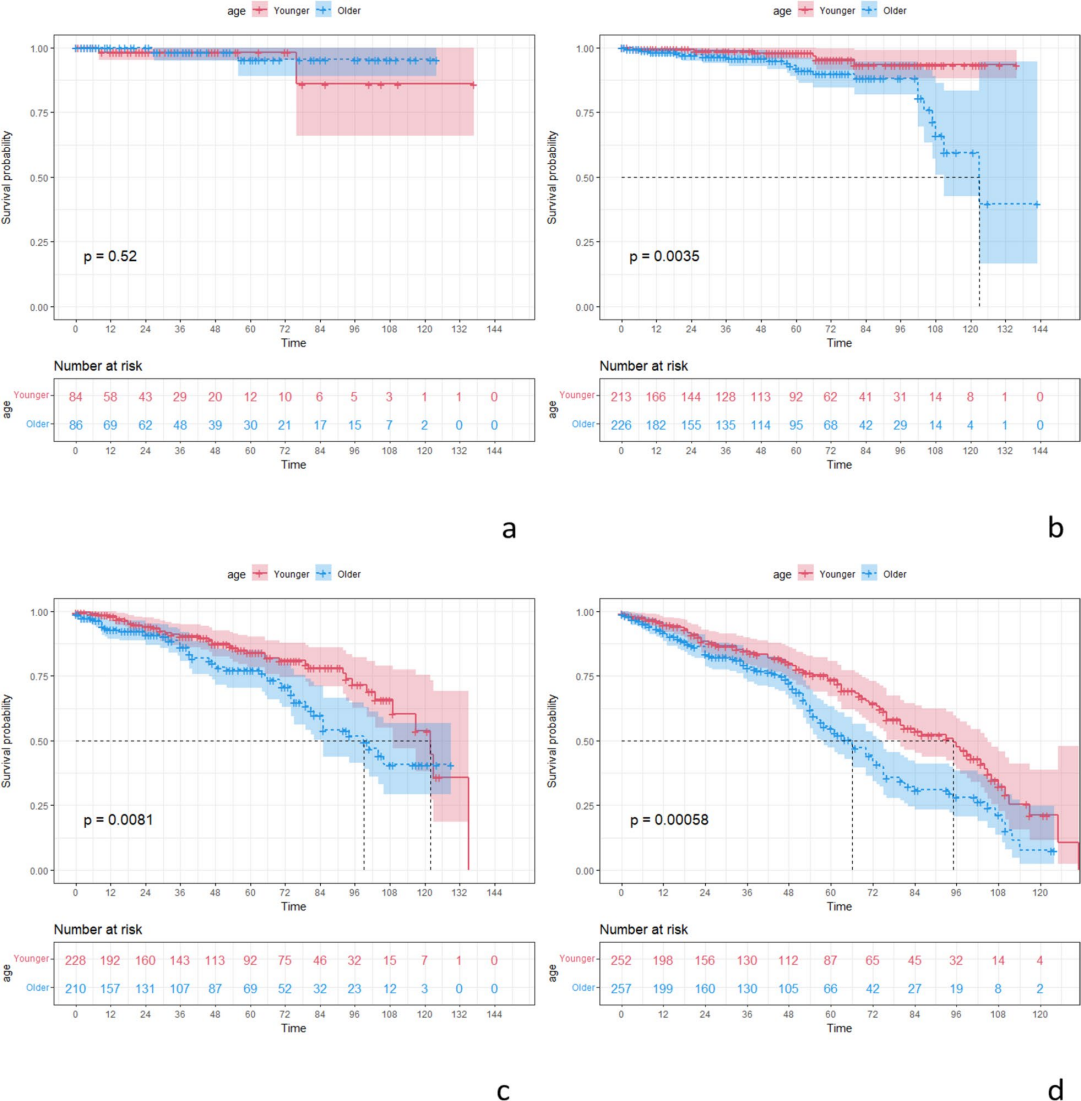
DSS curves of different age groups in different tumor pathological stages. **a** Stage I. **b** Stage II. **c** Stage III. **d** Stage IV

After PSM, the younger group had better DSS compared to the older group (Fig. 2). Regarding DSS at varied time points, the younger group had better 1-year DSS (*p* = 0.024) and 5-year DSS (*p* < 0.001) relative to the older group. For DSS in different tumor pathological stages, the younger group had better DSS in stage II (*p* = 0.0035), stage III (*p* = 0.0081), and stage IV (*p* < 0.001) compared to the older group (Fig. 4). For stage II CRC, the younger group had better 5-year DSS (*p* = 0.036) and 10-year DSS (*p* = 0.002); for stage III CRC, the younger group had better 1-year DSS (*p* = 0.022); and for stage IV CRC, the younger group had better 5-year DSS (*p* = 0.001).

## Discussion

This is a study based on a large CRC database from West China Hospital of Sichuan University, covering people from all over China, so the sample has good regional representativeness. For the samples obtained after strict inclusion and exclusion, we used an algorithm based on the Cox proportional hazards regression model to group age, and on this basis, we analyzed the difference in postoperative prognosis between young and older patients. To our knowledge, this is the first attempt in this field.

An aggregate of 2374 patients was encompassed within this study, including 1335 in the younger cohort and 1039 in the older cohort. After PSM, 784 patients from each group were enrolled at a 1:1 ratio. No significant differences were observed in the baseline data after PSM implementation. The multivariate analysis results revealed that older age was an independent risk factor for both OS and DSS. Overall OS and DSS were better in the younger group compared to the older group after PSM. In different tumor pathological stages after PSM, both OS and DSS were better in the younger group than in the older group for stages II, III, and IV CRC. The majority of OS and DSS at each time point in the younger group were better than those of the older group when comparing different age groups.

The effect of age on long-term survival in CRC patients remains a contentious issue. Zhao et al. [8] reported that younger age constitutes an independent risk factor for OS and DSS, with older patients demonstrating better OS and DSS outcomes. In addition, several studies reported no significant differences in OS or DSS between younger and older groups [20–22]. Nakayama et al. [10] proposed that younger patients exhibit equivalent or improved prognoses compared to their older counterparts. Wang et al. [23] reported that younger patients display better DSS. Liu et al. [24] reported that older age constitutes an independent risk factor for OS, with younger patients exhibiting better OS.

It is crucial to recognize that disparate methods of dividing age groups may impact the results when comparing the prognoses of younger and older patients. Previous studies have employed different cut-off ages, such as 35, 40, 44, 45, and 50 years old, frequently consulting established guidelines [8, 10, 20–24]. Notably, 50 and 45 years old are often selected as the cut-off ages, at which the American Cancer Society (ACS) recommends CRC screening [12]. These are based on United States-centric models, whereas some others have been developed globally [25, 26], rendering it potentially inappropriate to directly adopt cut-off ages from specific previous studies or guidelines. In the present study, utilizing the optimal cut-off age determined by the Cox proportional hazards regression model for age group division, based on the incorporated patient data, might be more suitable. The discovery of improved long-term survival in younger CRC patients under this age group division, which was inconsistent with our clinical observation that young patients with colorectal cancer may have a worse prognosis, may offer novel insights for further elucidating the association between age and prognosis in CRC patients. We may consider whether more intensive comprehensive treatments can bring better long-term prognosis to young patients with colorectal cancer. At the same time, suggestions can also be made for adjusting treatment plans for elderly patients.

For treatment, this may suggest that younger CRC patients may benefit better from CRC resection with preoperative and postoperative treatment in longer treatment cycles and higher intensity. Therefore, young CRC patients should have a more open attitude toward CRC resection. As for the specific age dividing line, perhaps it should be determined in a way that is suitable for local patients. Through an algorithm, we obtained a cut-off age, which might provide partial reference for surgical decision-making for patients of different ages, but its representativeness need to be confirmed by studies with larger samples and in more centers.

What needs to be clarified is that even so, the surgical benefits of older CRC patients should not be completely ignored. Turri et al. [27] thought that the preoperative identification of risk factors for low OS may help the selection of those old patients who may benefit from curative CRC surgery. Willemsen et al. [28] found that a considerable number of octogenarian colorectal cancer patients can still achieve 5-year survival after surgery. Perhaps we can also refer to the postoperative management of gastric cancer found by Qiu et al. [29], that is, to develop personalized follow-up strategies according to age and postoperative time, in order to detect recurrence as early as possible and decide on further treatment, so as to control DSS in old CRC patients.

Previous studies on the relationship between age and prognosis in CRC patients in particular tumor pathological stages have revealed analogous yet not identical results compared to the current study [23, 24]. These findings, on one hand, suggested that younger patients are inclined to exhibit better prognosis than older patients across various tumor pathological stages and, on the other hand, suggested that tumor pathological stage may be a critical independent predictor of prognosis in CRC patients. In fact, the multivariate analysis in this study indicated that tumor pathological stage was the largest independent predictor of OS, barring age, and the primary independent predictor of DSS. Consequently, it would be worthwhile to further explore the association between age and prognosis in CRC patients across different tumor pathological stages.

The multivariate analysis in this study revealed that besides the two most crucial factors—age and tumor pathological stage—tumor differentiation and surgical characteristic also function as independent predictors of both OS and DSS. Tumor stage remains the most pivotal prognostic factor in CRC; more advanced tumor stages and inferior morphological factors, such as lower tumor differentiation, typically signify poorer prognoses [30]. Poorer surgical characteristic entail greater residual tumor presence, resulting in more restricted improvements in tumor morphology, which may contribute to less favorable prognoses.

Regarding the elucidation of the relationship between age and prognosis in CRC patients in this study, the evidence remains circumscribed. In comparison to younger CRC patients, older CRC patients tend to exhibit concomitant diseases, such as chronic conditions, and older patients with concomitant diseases may be associated with inferior OS outcomes [31]. For early-onset CRC, despite enhanced treatment adherence and elevated treatment intensity in younger patients, disease biology is more unfavorable, characterized by advanced tumor stages, lower cell differentiation, and heightened prevalence of signet-ring cell carcinoma [32, 33]. However, younger people make up only a small proportion of CRC patients. Therefore, on a population basis, their prognosis may be more favorable than their older counterparts when controlling for disease, patient, and treatment factors [34]. It is well known that older patients are less tolerant of chemotherapy, which may also not conducive to prognosis. Furthermore, it is imperative to study whether additional age-related factors may influence the prognosis of CRC patients. There may be many reasons why the prognosis of young colorectal cancer patients is better than old ones, including nonspecific and specific reasons and direct and indirect reasons.

## Conclusion

The study revealed that, in comparison to their older counterparts, younger CRC patients may exhibit better long-term survival outcomes after surgery, particularly better OS and DSS for stages II, III, and IV CRC. Therefore, younger CRC patient may gain greater benefit from CRC resection and combined therapy. As for the cut-off age, it may be determined by a specific model suitable for local patients. Nonetheless, to substantiate these findings, it is imperative to conduct a multicenter cohort encompassing a more expansive sample size. Moreover, it is essential to undertake interventional studies to ascertain whether potential factors could ameliorate the long-term prognosis of older CRC patients.

## Data Availability

The data presented in this study are available on request from the corresponding author. The data are not publicly available due to data security.

## Abbreviations

CRC: Colorectal cancer
PSM: Propensity score matching
DACCA: Database from Colorectal Cancer
IARC: International Agency for Research on Cancer
OS: Overall survival
DSS: Disease-specific survival
R0: Complete resection under the naked eye
R1: Residual cancer under the microscope
R2: Residual cancer under the naked eye
BMI: Body mass index
SPSS: Statistical Product and Service Solutions

## References

[1] Sung H, Ferlay J, Siegel RL, Laversanne M, Soerjomataram I, Jemal A (2021). Global cancer statistics 2020: GLOBOCAN estimates of incidence and mortality worldwide for 36 cancers in 185 countries. Ca-a Cancer Journal for Clinicians.

[2] Morgan E, Arnold M, Gini A, Lorenzoni V, Cabasag CJ, Laversanne M (2023). Global burden of colorectal cancer in 2020 and 2040: incidence and mortality estimates from GLOBOCAN. Gut.

[3] Sun D, Li H, Cao M, He S, Lei L, Peng J (2020). Cancer burden in China: trends, risk factors and prevention. Cancer Biol Med.

[4] Feng RM, Zong YN, Cao SM, Xu RH (2019). Current cancer situation in China: good or bad news from the 2018 global cancer statistics?. Cancer Commun.

[5] Cao W, Chen HD, Yu YW, Li N, Chen WQ (2021). Changing profiles of cancer burden worldwide and in China: a secondary analysis of the global cancer statistics 2020. Chin Med J.

[6] Sung JJY, Chiu HM, Jung KW, Jun JK, Sekiguchi M, Matsuda T (2019). Increasing trend in young-onset colorectal cancer in Asia: more cancers in men and more rectal cancers. Am J Gastroenterol.

[7] Patel G, Patil P (2022). Worrisome trends in young-onset colorectal cancer: now is the time for action. Indian J Surg Oncol.

[8] Zhao L, Bao F, Yan J, Liu H, Li T, Chen H (2017). Poor prognosis of young patients with colorectal cancer: a retrospective study. Int J Colorectal Dis.

[9] Shida D, Ahiko Y, Tanabe T, Yoshida T, Tsukamoto S, Ochiai H (2018). Shorter survival in adolescent and young adult patients, compared to adult patients, with stage IV colorectal cancer in Japan. BMC Cancer.

[10] Nakayama Y, Kobayashi H, Kawamura H, Matsunaga R, Todate Y, Takano Y (2020). The long-term outcomes in adolescent and young adult patients with colorectal cancer -a multicenter large-scale cohort study. J Cancer.

[11] Perrott S, Laurie K, Laws K, Johnes A, Miedzybrodzka Z, Samuel L (2020). Young-onset colorectal cancer in the north east of Scotland: survival, clinico-pathological features and genetics. BMC Cancer.

[12] Wolf AMD, Fontham ETH, Church TR, Flowers CR, Guerra CE, LaMonte SJ (2018). Colorectal cancer screening for average-risk adults: 2018 guideline update from the American Cancer Society. CA Cancer J Clin.

[13] Willauer AN, Liu Y, Pereira AAL, Lam M, Morris JS, Raghav KPS (2019). Clinical and molecular characterization of early-onset colorectal cancer. Cancer.

[14] Taylor JMG, Yu MG (2002). Bias and efficiency loss due to categorizing an explanatory variable. J Multivar Anal.

[15] Printz C. American Cancer Society updates its colorectal cancer screening guideline: new recommendation is to start screening at age 45 years. Cancer. 2018;124(18):3631–2. 10.1002/cncr.31742. 10.1002/cncr.31742 30387885

[16] Burnett-Hartman AN, Lee JK, Demb J, Gupta S (2021). An update on the epidemiology, molecular characterization, diagnosis, and screening strategies for early-onset colorectal cancer. Gastroenterology.

[17] Steyerberg EW, Moons KG, van der Windt DA, Hayden JA, Perel P, Schroter S (2013). Prognosis Research Strategy (PROGRESS) 3: prognostic model research. PLoS Med.

[18] Barrio I, Xose Rodriguez-Alvarez M, Meira-Machado L, Esteban C, Arostegui I (2017). Comparison of two discrimination indexes in the categorisation of continuous predictors in time-to-event studies. SORT.

[19] Austin PC (2011). An introduction to propensity score methods for reducing the effects of confounding in observational studies. Multivariate Behav Res.

[20] Yang Z, Kang L, Wang L, Xiang J, Cai G, Cui J (2012). Characteristics and long-term survival of colorectal cancer patients aged 44 years and younger. Clin Transl Oncol.

[21] Wong SW, Ling DY, Yeow RQ, Chong RW, Aziz MRA, Aziz NA (2021). Clinicopathological patterns and survival outcomes of colorectal cancer among young adults in Malaysia: an institutional cohort study. Singapore Med J.

[22] McClelland PH, Liu T, Ozuner G (2022). Early-onset colorectal cancer in patients under 50 years of age: demographics, disease characteristics, and survival. Clin Colorectal Cancer.

[23] Wang L, Hirano Y, Heng G, Ishii T, Kondo H, Hara K (2020). Better cancer-specific survival in younger patients with stage III colorectal cancer: a propensity score matching study from Japan. Anticancer Res.

[24] Liu XY, Kang B, Cheng YX, Yuan C, Tao W, Zhang B (2022). The short-term and oncologic outcomes of younger vs older colorectal cancer patients undergoing primary surgery: a propensity score matching analysis. BMC Cancer.

[25] Ladabaum U, Dominitz JA, Kahi C, Schoen RE (2020). Strategies for colorectal cancer screening. Gastroenterology.

[26] Kastrinos F, Kupfer SS, Gupta S (2023). Colorectal cancer risk assessment and precision approaches to screening: Brave New World or Worlds Apart?. Gastroenterology.

[27] Turri G, Caliskan G, Conti C, Martinelli L, De Giulio E, Ruzzenente A (2022). Impact of age and comorbidities on short- and long-term outcomes of patients undergoing surgery for colorectal cancer. Front Oncol.

[28] Willemsen P, Devriendt S, Heyman S, Van Fraeyenhove F, Perkisas S (2023). Colorectal cancer surgery in octogenarians: real-world long-term results. Langenbecks Arch Surg.

[29] Qiu WW, Chen QY, Zheng WZ, He QC, Huang ZN, Xie JW (2022). Postoperative follow-up for gastric cancer needs to be individualized according to age, tumour recurrence pattern, and recurrence time. Eur J Surg Oncol.

[30] Zlobec I, Lugli A (2008). Prognostic and predictive factors in colorectal cancer. J Clin Pathol.

[31] McCleary NJ, Zhang S, Ma C, Ou FS, Bainter TM, Venook AP (2022). Age and comorbidity association with survival outcomes in metastatic colorectal cancer: CALGB 80405 analysis. J Geriatr Oncol.

[32] Mauri G, Sartore-Bianchi A, Russo AG, Marsoni S, Bardelli A, Siena S (2019). Early-onset colorectal cancer in young individuals. Mol Oncol.

[33] Fontana E, Meyers J, Sobrero A, Iveson T, Shields AF, Taieb J (2021). Early-onset colorectal adenocarcinoma in the IDEA database: treatment adherence, toxicities, and outcomes with 3 and 6 months of adjuvant fluoropyrimidine and oxaliplatin. J Clin Oncol.

[34] McKay A, Donaleshen J, Helewa RM, Park J, Wirtzfeld D, Hochman D (2014). Does young age influence the prognosis of colorectal cancer: a population-based analysis. World J Surg Oncol.

